# Sodium butyrate impedes the lymphoma caused by Marek’s disease virus via regulating the mitochondrial apoptosis pathway

**DOI:** 10.3389/fvets.2024.1360878

**Published:** 2024-02-28

**Authors:** Qiaoer Lin, Jun Zhou, Fan Yang, Congsen Zheng, Meiting Chen, Chuanzhe Chang, Shikai Cai, Feng Wen, Nina Wang, Yanfeng Chen, Limei Qin

**Affiliations:** ^1^School of Life Science and Engineering, Foshan University, Foshan, China; ^2^Guangdong Provincial Key Laboratory of Animal Molecular Design and Precise Breeding, Foshan University, Foshan, China

**Keywords:** sodium butyrate, lymphoma, apoptosis, mitochondrion, MDV, latency, *ICP4*

## Abstract

Sodium butyrate (NaB) has garnered attention in recent years for its ability to impede the malignant progression of tumors. In order to explore the potential inhibitory effects of NaB on the replication of Marek’s disease virus (MDV) and subsequent lymphoma formation, newly hatched chickens were infected with the vvMDV Md5 strain and administered NaB prior to (prevention group) or following (treatment group) Md5 inoculation. The results revealed that NaB played a pivotal role in diminishing both the incidence and fatality rates in chickens afflicted with Md5 infection. Notably, NaB exhibited a remarkable capacity to inhibit the expression of MDV immediate early genes, i.e., *ICP4* and *ICP27*, thus attenuating tumorigenesis in the chicken spleen. To further elucidate the mechanism of NaB on lymphoma cells, MDV bearing lymphoma cells, i.e., MSB-1 were exposed to NaB for 24 h prior to various experimental tests. The results revealed that NaB effectively hindered the proliferation, migration, and colony formation of MSB-1 cells. Furthermore, NaB demonstrated the ability to modulate the key molecules in mitochondrial apoptosis pathway. Taken together, these findings reveal that NaB can impede the lymphoma caused by MDV via regulating the mitochondrial apoptosis pathway, both *in vitro* and *in vivo*. These results suggest that the utilization of NaB warrants serious consideration as a promising approach for the prevention of MDV.

## 1 Introduction

Marek’s Disease (MD) is a highly contagious lymphoproliferative and immunosuppressive disease caused by the oncogenic α-herpesvirus Marek’s Disease Virus (MDV) (1). MD has caused significant economic losses to the global poultry industry over the past few decades. Although the use of vaccines has greatly reduced the incidence of MD, there has been a recent increase in reports of MD worldwide in the last few years (2–6). The disease is primarily transmitted through airborne dissemination of dust particles containing infected chicken feather follicle epithelial cells, which are inhaled by susceptible birds (7). In addition to causing mortality and reduced productivity in chickens, MD also induces immunosuppression in the host, making them susceptible to secondary infections by other pathogens (8).

MDV, a tumorigenic herpesvirus, is frequently utilized as a tool to investigate the pathogenesis of viral infections (9). Initiation of the viral process occurs when avian hosts respire airborne particles laden with viral entities, leading to the establishment of latency within CD4+ T cells in chickens and subsequent transformation (10). The successful establishment of latency confers the ability to perpetuate the viral genome, and the capacity to establish enduring latency within the host, with intermittent reactivation, represents a pivotal aspect of the virus’ survival strategy (11). Viral latency culminates in the infection of a substantial population of CD4+ T lymphocytes, while reactivation engenders the conversion of T lymphocytes into lymphoma cells that disseminate across various organs within the body (12). Consequently, the phases of latent infection and reactivation assume critical roles as pivotal junctures in the prevention and management of early MDV infection. The involvement of *ICP4* and *ICP27* in the progression of latent MDV infection contributes to the establishment and sustenance of latency. *ICP4* assumes a pivotal function in modulating the cascade of gene expression that governs viral infection. It engenders the transcription of MDV-specific genes in conjunction with multiple transcription factors, thereby promoting MDV replication and viral infection (13). *ICP27* is indispensable for viral replication, as it fosters early viral gene expression and DNA synthesis during productive infection (14). Obviously, the latent infection and reactivation phases are crucial stages in preventing and controlling MDV infection during the early stages. These two key periods provide an opportunity to extensively investigate the molecular mechanism of MDV infection and find solutions for the current challenges associated with MD.

Sodium butyrate (NaB), a sodium salt derivative of butyric acid, has exhibited the ability to trigger programmed cell death, known as apoptosis, in various leukemia cell lines, including U937 (15) and HL-60 cell lines (16). Furthermore, recent investigations have unveiled the capacity of NaB to impede cell proliferation (17), induce apoptosis (18), and facilitate cell differentiation (19) through the stimulation of histone hyperacetylation in cancer cells. Notably, both butyrate and propionate have demonstrated growth-inhibiting properties against colorectal adenocarcinoma cells (20), along with a pronounced enhancement of apoptosis in cancer cells (21). Similarly, in the context of breast cancer cells, NaB treatment has been observed to induce cell cycle arrest and foster apoptosis (22–24). In addition to the use of NaB in *in vitro* experiments, butyrate administration in mouse models has been found to diminish the occurrence of colon tumors induced by carcinogens, while also eliciting apoptosis in tumor cells (25). Within the realm of poultry research, investigations primarily focus on exploring the effects of NaB on production performance, gut microbiology (26), and its potential benefits in addressing gastrointestinal diseases in poultry (27, 28). However, the impact of NaB on poultry viral diseases remains an area that warrants further exploration and understanding.

Given the importance of exploring the mechanism of lymphoma caused by MDV and the advantages of the role of NaB in promoting apoptosis of tumor cells, in this study, NaB was investigated *in vivo* and *in vitro* by using the MDV infected chicken model and MDV-transformed lymphoma cell, i.e., MSB-1, respectively.

## 2 Materials and methods

### 2.1 Ethics statement

All procedures involving animals in this research were conducted in accordance with the ethical standards of the institution or practice. The experiments were approved by the Ethics Committee for Animal Experiments of Foshan University (Ethical permit number: FOSU2008).

### 2.2 Virus and cells

The highly pathogenic MDV Md5 strain and the MDV-transformed T-cell line MSB-1 (in suspension) were cultivated in our laboratory. The cells were nurtured in RPMI-1640 medium (Servicebio, China), supplemented with 10% FBS (Gibco, United States) and 1% antibiotic solution (penicillin 100 U/mL, streptomycin 100 g/mL; Beyotime, Jiangsu, China), and incubated at 37°C in a 5% CO_2_ environment. Chicken embryonic fibroblasts (CEFs) were prepared from 9 to10-day-old specific pathogen-free (SPF) chicken embryos using conventional methods.

### 2.3 CCK-8 assay

Cell Cytotoxicity/Viability Assay were performed using the CCK-8 Cell Counting Kit (Vazyme, Nanjing, China) in accordance with the manufacturer’s instructions as described previously (29). MSB-1 cells in the logarithmic growth phase were seeded in 96-well plates at a density of 5 × 10^3^ cells per well. Subsequently, the cells were exposed to various concentrations of NaB (0, 2, 4, 6, 8, and10 mM) for a duration of 24 h. The subsequent day, 10 μL of CCK-8 solution were introduced, followed by an additional incubation period of 2 h. Concurrently, the control cells were subjected to RPMI-1640 medium supplemented with 10% CCK-8.

### 2.4 Plaque assay

Following the previously described method with some modifications (29, 30), plaque assays were performed. Once the monolayer of CEF cells in a 96-well tissue culture plate reached confluency, the treatment group was infected with 20 PFU of the Md5 virus per well for 1 h. After replacing the infection medium, NaB solution was added to a final concentration of 6 mM, and the cells were incubated for 24 h. Subsequently, the cells were covered with a 2% agarose gel containing an equal volume of 2× DMEM medium. For the prevention group, CEF cells were incubated with NaB solution (final concentration of 6 mM) for 24 h before viral infection, followed by the same infection protocol as the treatment group. The agarose gel was then applied. The number of cytopathic effect (CPE) lesions was observed daily and counted until no new CPEs were observed.

### 2.5 Experimental design

Local chicken Nanhai Ephedra chickens, devoid of any prior immunization, were procured from Nanhai Breeder Co. The chickens did not receive any vaccination procedures throughout the experiment. The NaB reagent was obtained from Sigma-Aldrich Chemical Company (303,410, Sigma Aldrich, Germany). Based on the drug dosage mentioned in the referenced literature (31) and subsequent conversions (32) of drug dosages, the approximate administration doses of the drug in the experiment were calculated using conversion factors for dose equivalence between chickens and other experimental animals (33). Building upon preliminary investigations, NaB was administered at a dose of 132 mg/kg.

**Figure 1:**
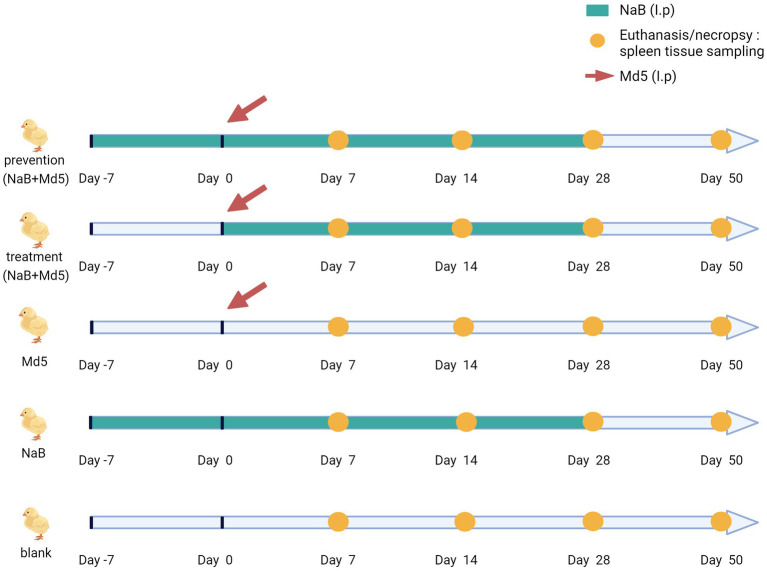
Animal experimental design.

To ensure adequate and consistent absorption of NaB in the chickens, intraperitoneal injection was employed during the experiment. In the three groups of NaB, prevention, and treatment, chickens of each group were injected with 100 μL of NaB solution. The groups of NaB and prevention were received the NaB injection 7 day in advance (day −7), the duration of NaB administration was 35 days in total. The group of treatment was received the NaB injection at the same time as MD5 infection (day 0), the duration of NaB administration was 28 days in total (Figure 1). While the two groups of Blank and Md5 were injected with an equal volume of PBS solution. The chickens in each group underwent bi-daily weighing, allowing for necessary adjustments in the injected amount of NaB based on their weights. A total of 120 1-day-old chickens were randomly allocated into five groups (*n* = 24) and housed in separate enclosures. Animals infected with non-human infectious viruses are isolated from healthy animals throughout the experiment to prevent transmission. Intraperitoneal injection of Md5, 2,000 PFU/chicken, was administered to the chickens, as outlined in previous literature (34). At each time point of dissection, five chickens were randomly selected from each group for sampling. Each spleen sample was tested with five replicates. Control groups included the Md5 group, NaB group, and blank group. Following Md5 infection, chickens were monitored daily for manifestations of MD, such as leg paralysis, head and neck rotation, blindness, tumor formation, and mortality. At the conclusion of the experimental period, surviving chickens were euthanized in a humane manner and subjected to dissection, focusing on organs like the liver, spleen, kidney, and heart, to assess the presence of internal tumors. The spleen index was calculated according to the formula, spleen index = weight of spleen (g) / body weight (g) × 100 (30). All chickens, whether deceased or alive, displaying MD symptoms or visibly detectable tumors were classified as MD cases. Cases that succumbed prematurely due to compromised autoimmunity or environmental factors within a week prior to the disease onset were excluded from analysis. Throughout the duration of animal experiments, restricted access, record keeping, animal isolation, sanitation and disinfection, sample collection, waste management, etc. have followed the relevant biosafety regulations, as detailed in Supplementary File 1.

### 2.6 Sample collection and processing

On 7, 14, 28, and 50 days post-infections (dpi), Chicks from the respective experimental cohorts were humanely terminated through exsanguination via the ruptured jugular vein and used diazepam as a sedative drug. For each time point, a minimum of three chicks from each group were euthanized, and spleen tissues were meticulously procured. The spleens were carefully weighed, and the spleen index was ascertained by computing the ratio of spleen weight to body weight. The procured tissues were subsequently delicately placed within 1.5 mL sterile test tubes that were preloaded with 1 mL of RNA Keeper Tissue Stabilizer (R501, Vazyme, Jiangsu, China). According to the reagent instructions, the samples were incubated overnight at 4°C and stored at −80°C as previously described (35).

### 2.7 Histopathological observation

Spleen specimens were procured from chickens at various time intervals to facilitate the histopathological analysis. The specimens were meticulously preserved in a formaldehyde solution. Subsequently, the tissues underwent a process of alcohol dehydration, after which 5 μm sections of paraffin-embedded spleen tissue were meticulously stained with the classic hematoxylin and eosin (H&E) technique (36). Following staining, the sections were subjected to dehydration using anhydrous ethanol, and subsequently rinsed in xylene to enhance transparency. Ultimately, the transparent sections were meticulously embedded in a neutral resin medium and examined under a light microscope.

### 2.8 Fluorescence quantitative PCR

Total RNA was isolated from chicken spleen tissues utilizing TRIzol reagent (B511311, Sangon biotech, Shanghai, China). Subsequently, the RNA samples were subjected to reverse transcription using Hiscript® III All-in-One RT SuperMix Perfect for qPCR reverse transcriptase (Vazyme, Nanjing, China). Each sample was employed to evaluate the expression of MDV genes and host mitochondrial apoptosis pathway genes. The quantitative fluorescence detection instrument employed was the Thermo QuantStudio 5, and the qPCR reaction system consisted of a 20 μL volume. This system encompassed 10 μL of Hieff UNICON® Universal Blue qPCR SYBR Green Master Mix, 0.5 μL each of upstream and downstream primers (10 μmol/L), 1 μL of cDNA, and the remaining volume was adjusted to 20 μL using ddH_2_O. Following pre-denaturation at 95°C for 2 min, amplification was conducted for 40 cycles (95°C for 10 s, 60°C for 30 s). Each sample was tested in triplicate, and the data are presented as mean ± SD. Relative quantification of the genes was determined using the 2^−∆∆CT^ method. The primer sequences used are depicted in Table 1 (37, 38, 40).

**Table 1:** Sequences of qPCR primers.

Primers	Reverse (5′-3′)	Forward (5′-3′)
TNF-α (37)	ACTATCCTCACCCCTACCCTGTC	GGTCATAGAACAGCACTACGGG
FADD (38)	ACTGGAAAATGCTGATGCGA	CCACTCTCGAAGCGACTGAAA
TRADD (38)	ACAACAGAACGCTCACACCA	TCCCTCTCGGTCGTATTCGT
PARP	AAAACGTGCTGCTGAAGTGT	GCTCCAGGGATTCATTGGCT
MCL-1 (39)	GTGCCTTTGTGGCTAAACACT	AGTCCCGTTTTGTCCTTACGA
Bak	CGTCTACCAGCAAGGCATCA	ATTGTCCAGATCGAGTGCAGC
Caspase-8	TGGCATGGCTACTGTGGAAG	TGAGATCCTTGCGAAGTGGG
BCL2	AGAGCGTCAACCGGGAGAT	CCACAAAGGCATCCCATCCT
Caspase-3	ACTGTCATCTCGTTCAGGCA	TGCTTCGCTTGCTGTGATCT
Caspase-9	GCCTGTGGAGGAGAACAAAAG	GTCTGGCTCGTCCTCATTCC
ARRDC3	CGGCCACGAGAGAGATGATG	TTCACCCAATAGCGCACACT
BAX	GCACAGCTTTATCCGCAAGG	CGCACAGGTGAGACAAAGGT
CytC	AAATGTTCCCAGTGCCATACG	TTGTCCTGTTTTGCGTCCAA
Apaf-1	AAGGGCATAAGGAAGCAATC	CAGTTTTGTCAGCAGAAGTAG
Meq	CGTCTTACCGAGGATCCCGAA	TCCCGTCACCTGGAAACCAC
ICP27	TCCGATGAAAATGCCGAAGTGA	GGAATCAGGAGATGGTCGTTCGGTCAA
ICP4	CTATGGCAGACATCGCTCGT	GTATCGCATCCAAGCAGTCC
PTEN	GGAAAGGGACGAACTGGTGT	CCGCCACTGAACATGGGAAT
TGF-β	GGAGCTGTACCAGGGTTACG	AAGGAGAGCCACTCATCGTC
β-actin	GATATTGCTGCGCTCGTTGTT	ACCAACCATCACACCCTGA

### 2.9 TUNEL assay

Apoptosis was detected in the spleens of each group at 7 dpi using the TUNEL apoptosis detection kit (40306ES20, Yeasen, Shanghai, China) (39). Tissue sections were deparaffinized in xylene, followed by immersion in anhydrous ethanol for 5 min and sequential washes in gradient ethanol (90, 80, 70%) for 1 time each, with a duration of 3 min per wash. After rinsing the sections with PBS, proteinase K solution was added and incubated at room temperature for 20 min. Subsequently, the sections were washed with PBS and incubated with TDT enzyme, dUTP, and buffer solution. The sections were then incubated at 37°C without light for 60 min. After washing the samples three times with PBS, DAPI solution was applied to stain the cell nuclei for 10 min at room temperature. Following sample washing, fluorescence microscopy was used for examination and image acquisition. DAPI emits blue light with an excitation wavelength of 330–380 nm and an emission wavelength of 420 nm, while FITC emits green fluorescence with an excitation wavelength of 465–495 nm and an emission wavelength of 515–555 nm.

### 2.10 Transwell assay

To assess the impact of NaB on cellular migration, the MSB-1 cells were cultivated in the upper compartment of 8 μm Transwell plates, with a density of 10^5^ cells per well, and immersed in 200 μL of serum-free medium. In the lower compartment, a 10% FBS solution was carefully introduced. NaB was then introduced into the medium to reach a final concentration of 6 mM. The wells were subsequently incubated under optimal conditions of 37°C and 5% CO_2_ for a duration of 24 h. Following the incubation period, the cells were meticulously fixed with a formaldehyde solution for a period of 30 min. Subsequently, a 0.4% crystal violet solution was employed to stain the cells for a duration of 10 min at ambient temperature. To evaluate the efficacy of the treatment, five microscopic fields were randomly selected, and images were acquired utilizing a fluorescence microscope (Revolve FL, Echo Laboratories, United States) with a magnification of 100 × .

### 2.11 Soft agar colony formation

To assess the impact of NaB on MSB-1 clone formation, a colony formation assay in soft agar was conducted. The experiment was conducted according to the method described by Stanley Borowicz et al. (41). The procedure involved preparing the bottom gel layer by mixing 1.2% agar with 2× medium in equal volumes, which was then spread as the bottom layer in 6-well plates. The plates were incubated at 37°C for 30 min to allow the gel to solidify. Subsequently, 6 × 10^3^ MSB-1 cells were mixed with 0.7% agar and added to the plates as the upper gel layer. In the NaB group, a NaB solution was added to the upper gel layer to achieve a concentration of 6 mM. To prevent excessive drying, 100 μL of complete medium was added after solidification. After a two-week incubation period, the colonies were observed under a microscope, manually counted, and photographed for analysis.

### 2.12 Flow cytometry

To assess the impact of NaB on MSB-1 apoptosis, a flow cytometry assay was conducted. Cells were treated with or without NaB at a final concentration of 6 mM for 24 h. Subsequently, the cells were suspended and stained with Annexin V-FITC and propidium iodide (PI) according to the instructions provided with the Annexin V-FITC/PI Apoptosis Kit (Elabscience, China), as described by Han et al. (42). The resulting data were analyzed using Flow Jo V10 software for further interpretation.

### 2.13 Statistical analysis

The data from this experiment were presented as mean ± S.D. Statistical analysis was performed using SPSS 26.0 (SPSS Inc., Chicago, United States). The Morbidity and mortality were analyzed by SPSS Chi-Square comparing between each group and results are presented in Table 2. For normally distributed data, the experimental groups were compared using one-way ANOVA followed by the Tukey test. In the case of non-normally distributed data, the Dunnett’s T3 test was applied. The Dunnett’s T3 test was chosen because it is robust to the analysis of unequal variances (43). For the comparison of two treatments, the Student’s two-tailed t-test was employed. A significance level of *p <* 0.05 was considered statistically significant. Data visualization was accomplished by GraphPad software (CA, United States).

**Table 2:** Morbidity after attack by Md5 strain.

Groups	Total numbers	Diseased birds	Morbidity (%)	Deaths	Mortality (%)
NaB+Md5 (prevention group)	23	2	8.7%^a,b^	1	4.30%
Md5 + NaB (treatment group)	22	2	9.1%^a,b^	1	4.50%
Md5	24	7	29.2%^b^	2	8.30%
NaB	24	0	0%^a^	0	0%
Blank	24	0	0%^a^	0	0%

Total number = number of non-pathologically death. All the birds showing clinical MD symptoms, death and survivals with gross tumors were classified as MD cases. Morbidity (%) = number of MD cases / total number × 100. Mortality (%) = number of death / total number × 100. Different letter means significant difference (*p* < 0.05).

## 3 Results

### 3.1 Effect of NaB on cytotoxicity in CEF cells

**Figure 2:**
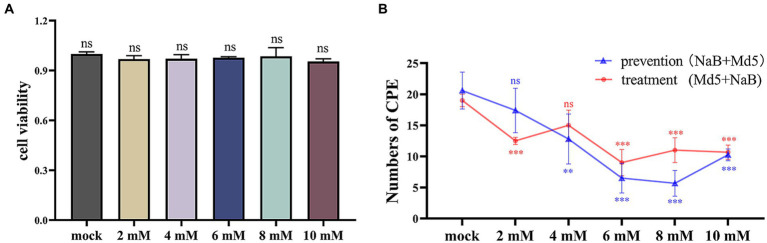
Cytotoxicity assay of CEF cells and statistics of CPE quantity. **(A)** The results of the cytotoxicity assay of CEF cells. **(B)** Statistical analysis of CPEs in plaque assay. *, * *, ***: statistically significant at *p* < 0.05, *p* < 0.01, *p* < 0.001 compared with mock group.

The safety concentration of NaB on CEF cells was evaluated using the CCK-8 assay as a preliminary step. As shown in Figure 2A, the cell viability was not affected in the experimental groups compared to the control group across different concentrations of NaB.

### 3.2 Effect of NaB on MDV replication in CEF cells

The impact of NaB on MDV replication was assessed using a plaque assay. As shown in Figure 2B, both the prevention and treatment groups exhibited a general trend where a higher concentration of NaB resulted in a lower number of CPE. In the prevention group, the lowest number of CPE was observed at the concentration of 8 mM, while in the treatment group, the lowest number of affected cells was observed at 6 mM.

### 3.3 Effect of NaB on mortality and morbidity in different groups

The tumor tissues were collected from each group and subjected to histological examination. Cumulative morbidity, mortality, and tumor incidence were evaluated for all groups of birds, except for those that died prematurely due to non-specific causes prior to the Md5 challenge. Compared to the Md5 group, both the prevention group and the treatment group that received intraperitoneal injection of sodium butyrate had lower tumor incidence and mortality rates (Table 2).

### 3.4 Effect of NaB on spleen lesions

**Figure 3:**
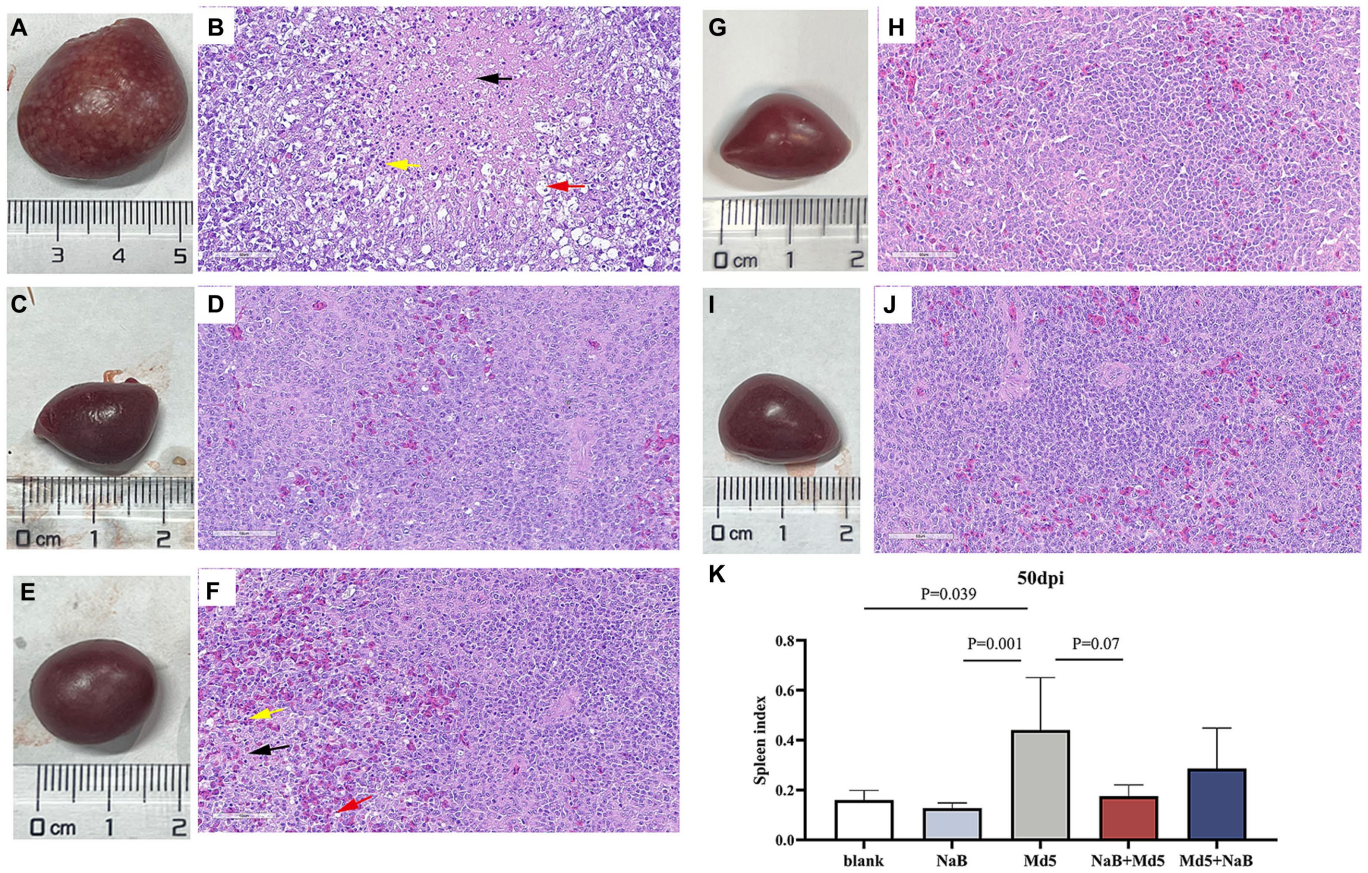
Histological lesions of spleen and index of spleen. Panels **(A,C,E,G,I)** show the spleens of the Md5 group, prevention group, treatment group, blank group and NaB group, respectively. Panels **(B,D,F,H,J)** show the sectioned images (40×) of each group. Black arrowheads indicate necrotic foci; Yellow arrowheads indicate inflammatory cell infiltration; Red arrowheads indicate reticular cell hyperplasia. Panel **(K)** shows the spleen index of each group at 50 dpi.

Necropsy was performed on all chickens to assess the presence of tumors in internal organs, with the findings depicted in Figure 3. At 50 dpi, the Md5 group exhibited visually detectable tumor lesions in the spleen, which displayed significant enlargement compared to the other groups (Figure 3A). The presence of tumorous lesions in the spleen of the Md5 group results in an increase in both the weight and volume of the spleen. Figure 3K demonstrates a difference in splenic index in each group. No significant difference in spleen index was observed among the prevention group, treatment group, and blank group, which indicates the NaB in prevention and treatment groups did not cause splenomegaly.

### 3.5 Effect of NaB on viral genes of MDV in spleen

**Figure 4:**
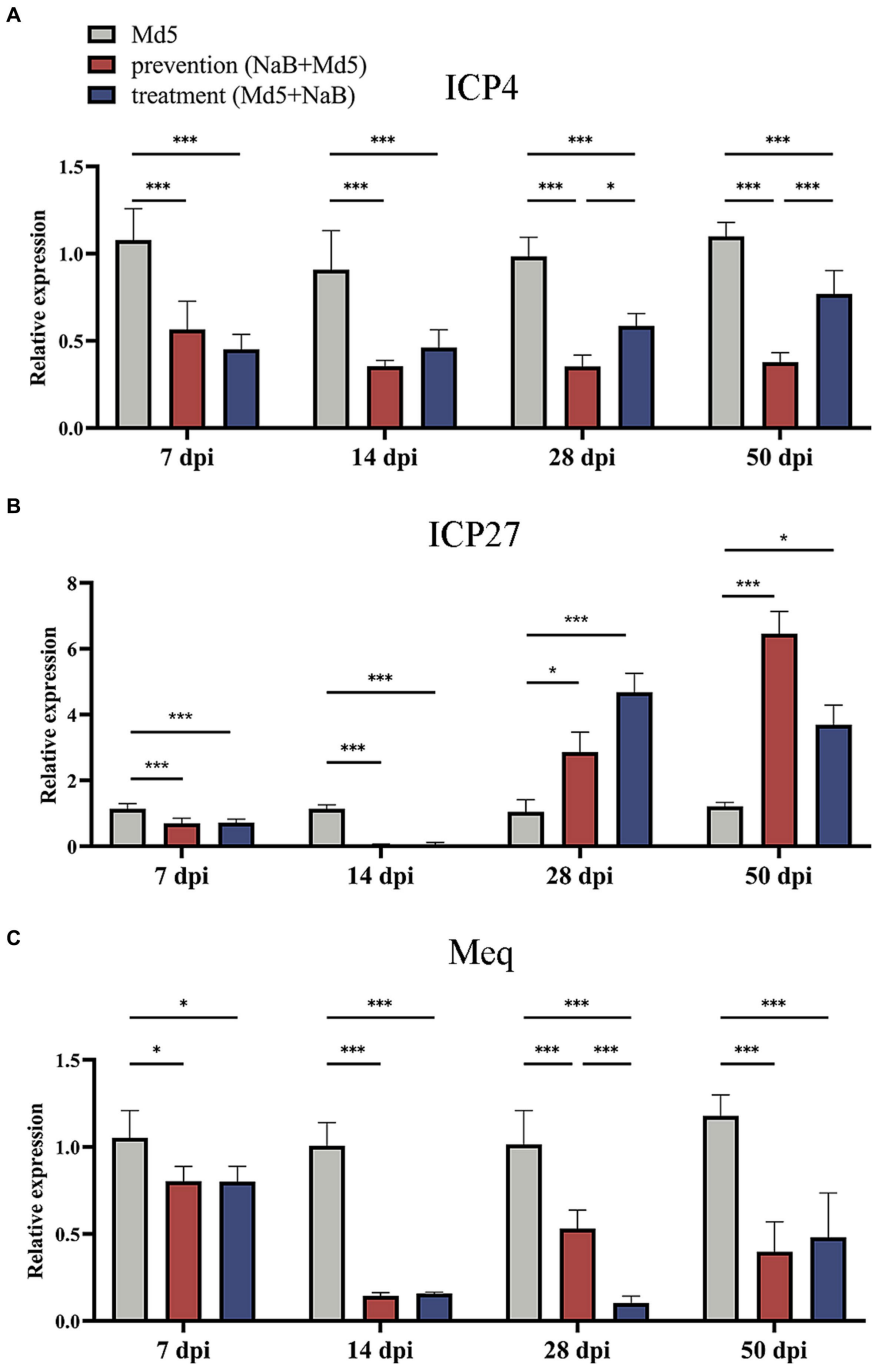
Expression of viral genes of MDV in the spleen. *, * *, ***: statistically significant at *p* < 0.05, *p* < 0.01, *p* < 0.001 between groups. **(A)** Relative expression of *ICP4*. **(B)** Relative expression of *ICP27*. **(C)** Relative expression of *Meq*.

The MDV immediate-early gene *ICP4* is a transcription activator that is an essential factor for early and late promoter for viral gene expression (44), while another immediate-early gene *ICP27* is known to be required for efficient expression of some viral DNA replication-related early genes and late viral genes as well as for virus growth (14). The quantification of *ICP4* and *ICP27*, along with MDV oncogene *Meq*, was assessed in the spleen using qPCR. Regarding the *ICP4* gene, treatment with NaB led to a substantial downregulation at all four time points in both the prevention and treatment groups, relative to the Md5 control group. Interestingly, at 28 dpi and 50 dpi, the prevention group displayed a significantly greater inhibition of *ICP4* expression compared to the treatment group, as illustrated in Figure 4A. Regarding the *ICP27* gene, no significant changes in expression were observed at 7 dpi among the three groups. However, a significant suppression was detected in both the prevention and treatment groups at 14 dpi, when compared to the Md5 control group. It is noteworthy that this suppression effect was reversed at 28 dpi and 50 dpi, as depicted in Figure 4B. In relation to the oncogene *Meq*, a notable reduction in relative expression was observed in both the prevention and treatment groups at 7, 14, 28, and 50 dpi, when compared to the Md5 control group. Furthermore, at 28 dpi, the treatment group exhibited a significantly more pronounced inhibition of *Meq* expression compared to the prevention group, as depicted in Figure 4C.

### 3.6 Effect of NaB on tumor-suppressive genes of spleen

**Figure 5:**
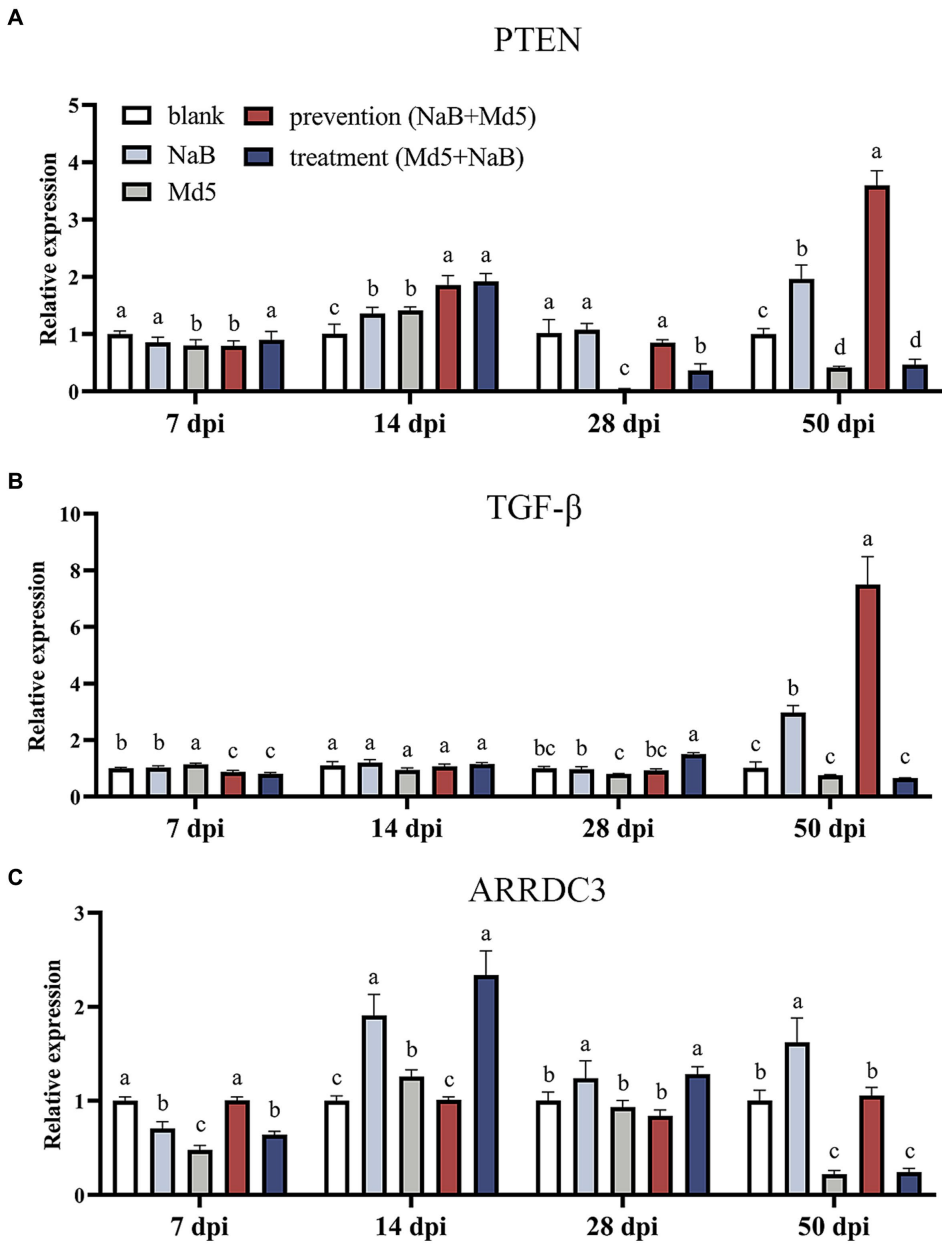
Expression of tumor suppressive genes in spleen. Different letter means significant difference (*p* < 0.05). **(A)** Relative expression of PTEN. **(B)** Relative expression of TGF-*β*. **(C)** Relative expression of *ARRDC3*.

Effect of NaB on tumor-suppressor genes in MD5 infected chicken spleen, such as *PTEN*, *TGF-β*, and *ARRDC3*, within each experimental group are depicted in Figure 5. Notably, a profound upregulation of *PTEN* was observed in the prevention group at 14, 28, and 50 dpi, as compared to the Md5 control group (Figure 5A). Furthermore, at 50 dpi, the expression of *TGF-β* surpassed that of the other three groups within the prevention group, indicating a potential role in tumor suppression (Figure 5B). Interestingly, the expression of *ARRDC3* exhibited a significant upregulation at both 7 and 50 dpi, highlighting its involvement in the prevention group when compared to the Md5 control group.

### 3.7 Effect of NaB on splenic apoptosis

**Figure 6:**
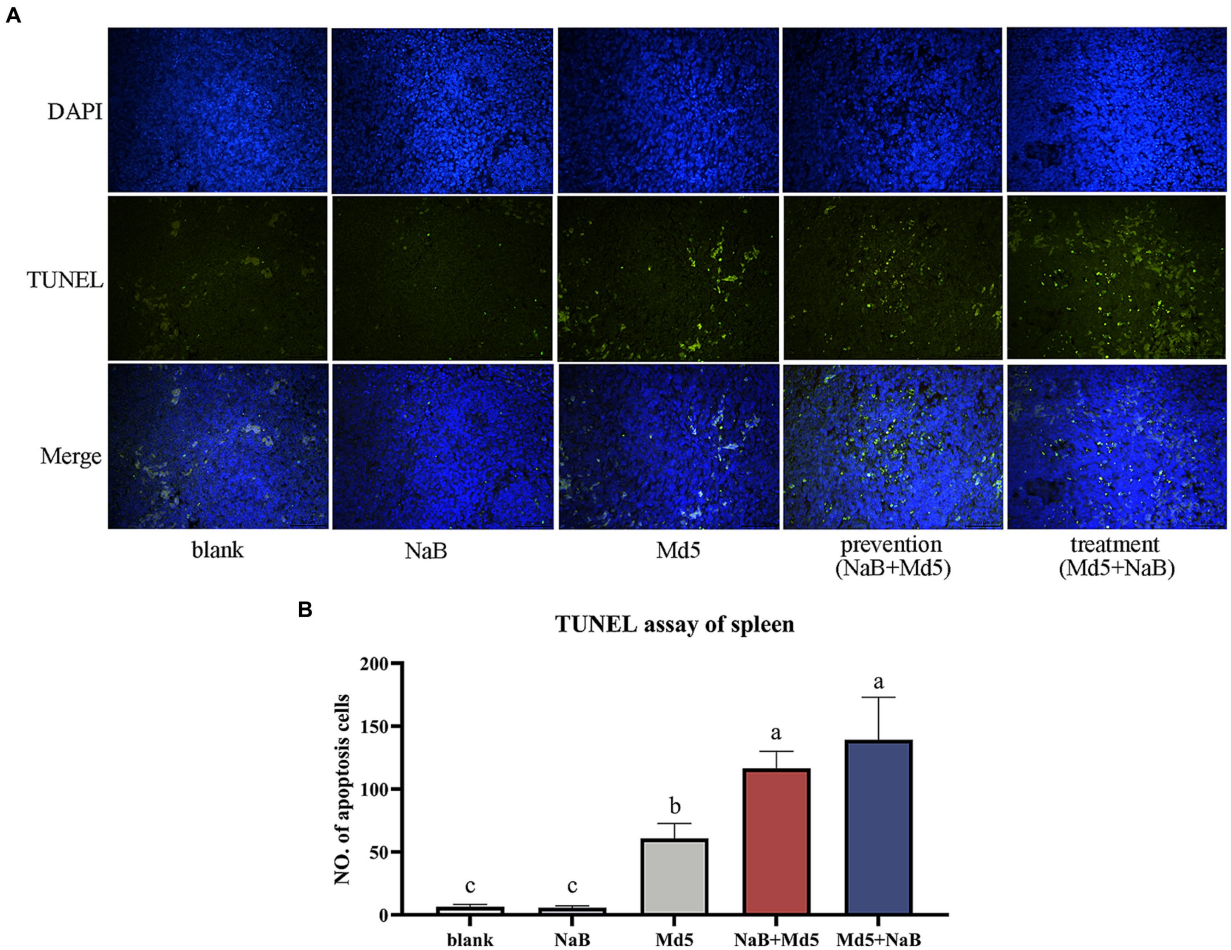
The TUNEL assay of spleen. **(A)** The DAPI (blue) and TUNEL (green) staining of each group. **(B)** The quantify of TUNEL assay of each group.

To explore the apoptosis in the spleen at 7 dpi after MDV infection, we conducted a TUNEL assay on spleen tissues. The results, as depicted in Figure 6, reveal a significant increase in green fluorescence signals within both the prevention and treatment groups, as compared to the Md5 group. Figure 6B further presents a quantitative representation of the extent of apoptosis in splenocytes. Remarkably, the treatment and prevention groups exhibited higher levels of apoptosis, followed by the Md5 group.

### 3.8 Effect of NaB on MSB-1 proliferation

**Figure 7:**
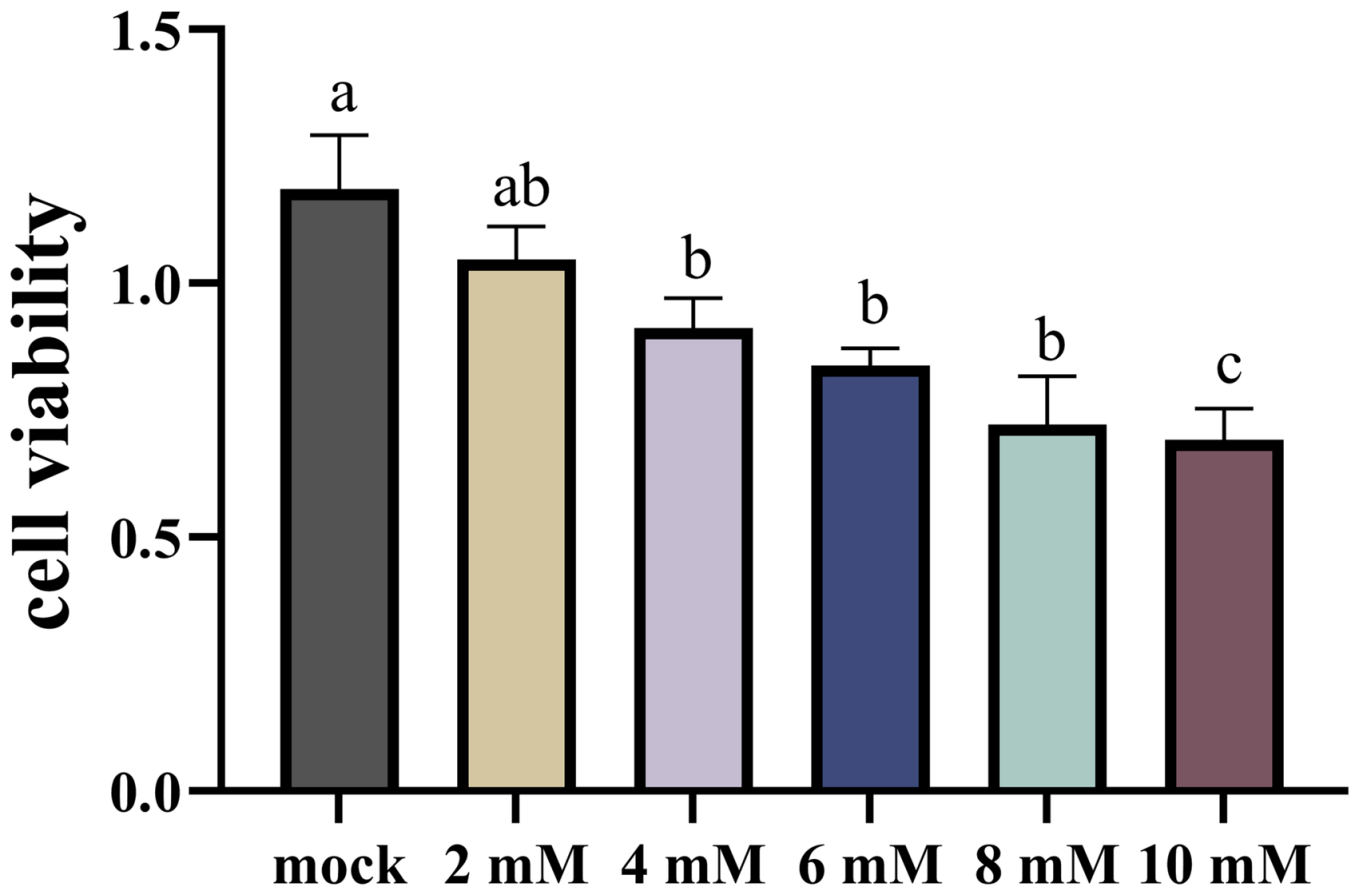
The CCK-8 assay on MSB-1 treated with NaB. Different letter means significant difference (*p* < 0.05).

In order to ascertain the suppressive impact of NaB on cellular proliferation, a cohort of MSB-1 cells were subjected to incubation with varying concentrations of NaB for a duration of 24 h. The outcomes of this experimental endeavor are depicted in Figure 7. Upon administration of a 2 mM concentration of NaB, no substantial alteration in the vitality of MSB-1 cells was observed. However, once the concentration of NaB reached or surpassed 4 mM, a propensity to impede the proliferation of MSB-1 cells became readily apparent.

### 3.9 Effect of NaB on MSB-1 migration and clone formation

**Figure 8:**
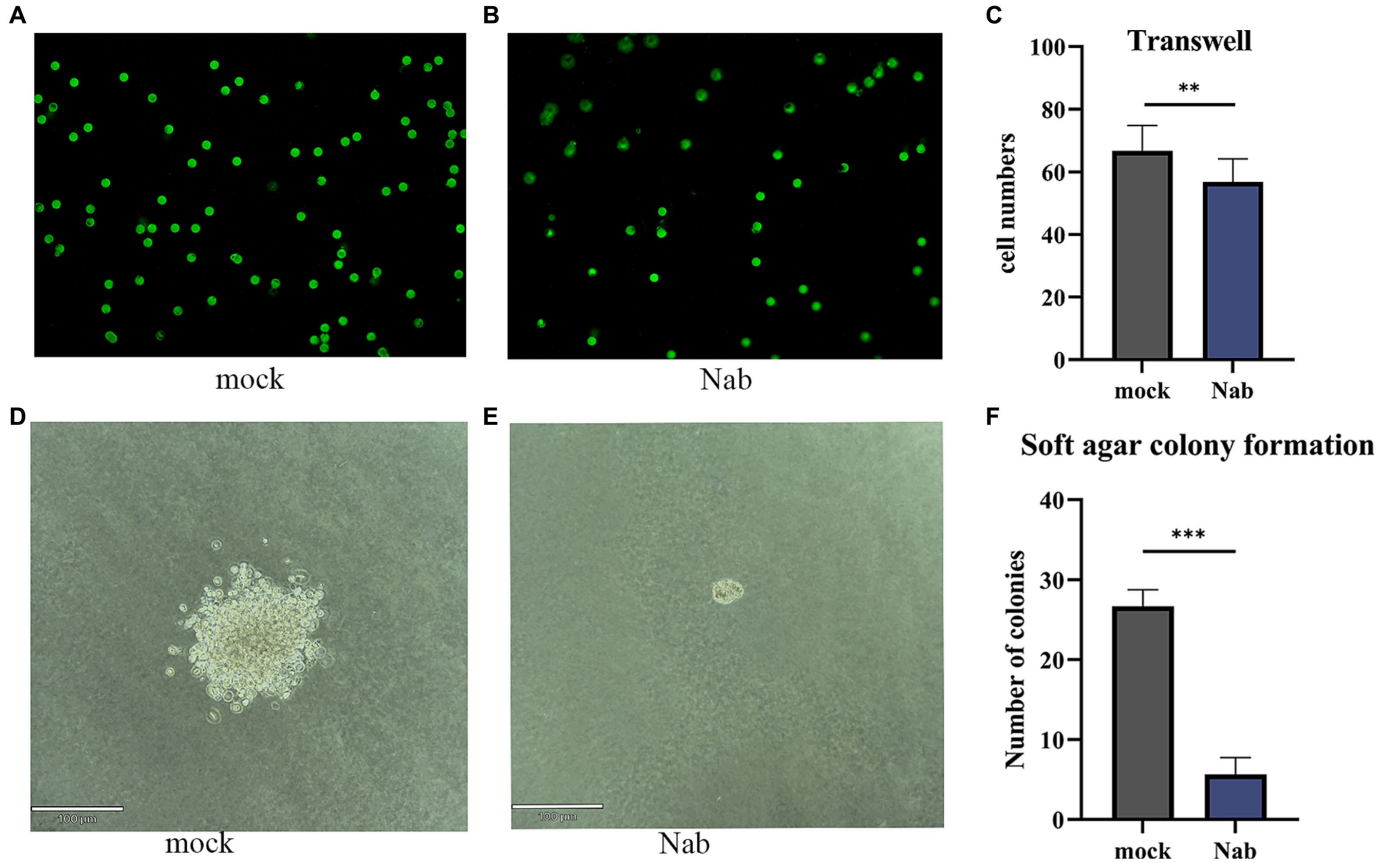
Transwell and soft agar cloning assays on MSB-1 cells. The migration of cells under a fluorescence microscope in the mock **(A)** and NaB group **(B)** and the cell clone formation in Mock group **(D)** and NaB group **(E)** were captured under a magnification of 200×. The number of migrated cells and clones were quantified respectively, statistical analyses were presented in **(C)** and **(F)**. *, * *, *** indicates the *p* < 0.05, *p* < 0.01 and *p* < 0.001, respectively.

In order to assess the impact of NaB on cellular migration, we conducted a Transwell assay on MSB-1 cells. Following a 24-h migration period, cells were stained and subsequently examined under a fluorescence microscope. Our observations revealed a noteworthy suppression of cell migration in the experimental group, wherein an additional 6 mM NaB was administered (Figures 8A–C). To further explore the influence of NaB on cellular migration, a soft agar colony assay was performed. Remarkably, the treatment of MSB-1 cells with NaB resulted in a considerable reduction in both clone size and cell count when compared to the control group (Figures 8D–F).

### 3.10 Effect of NaB on MSB-1 apoptosis

**Figure 9:**
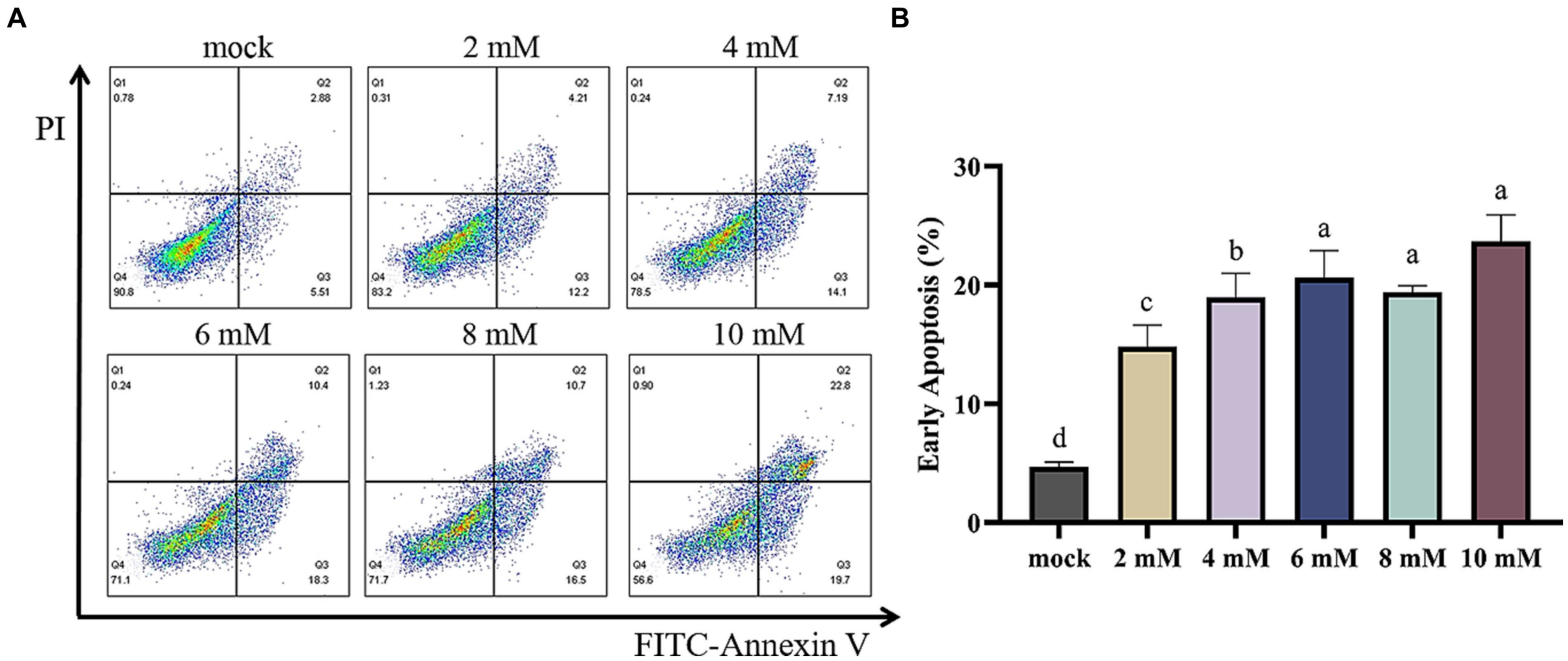
Flow cytometry of apoptosis in MSB-1 cell treated with different concentration of NaB. **(A)** Plots of apoptosis in MSB-1 cell via flow cytometry. **(B)** Quantitative analysis. Different letters above the columns mean there is significant difference (*p* < 0.05).

In order to assess the occurrence of programmed cell death in MSB-1 cells, a flow cytometry assay was conducted subsequent to the administration of various concentrations of NaB for a duration of 24 h. The concentrations tested ranged from 0 to 10 mM (Figure 9A). The resulting data was analyzed and represented graphically in Figure 9B, highlighting the distribution of cells within the quadrants Q1, Q2, Q3, and Q4. These quadrants are indicative of the quantities of dead cells, late apoptotic cells, early apoptotic cells and lived cells, respectively.

### 3.11 Effect of NaB on the mitochondrial apoptosis pathway

**Figure 10:**
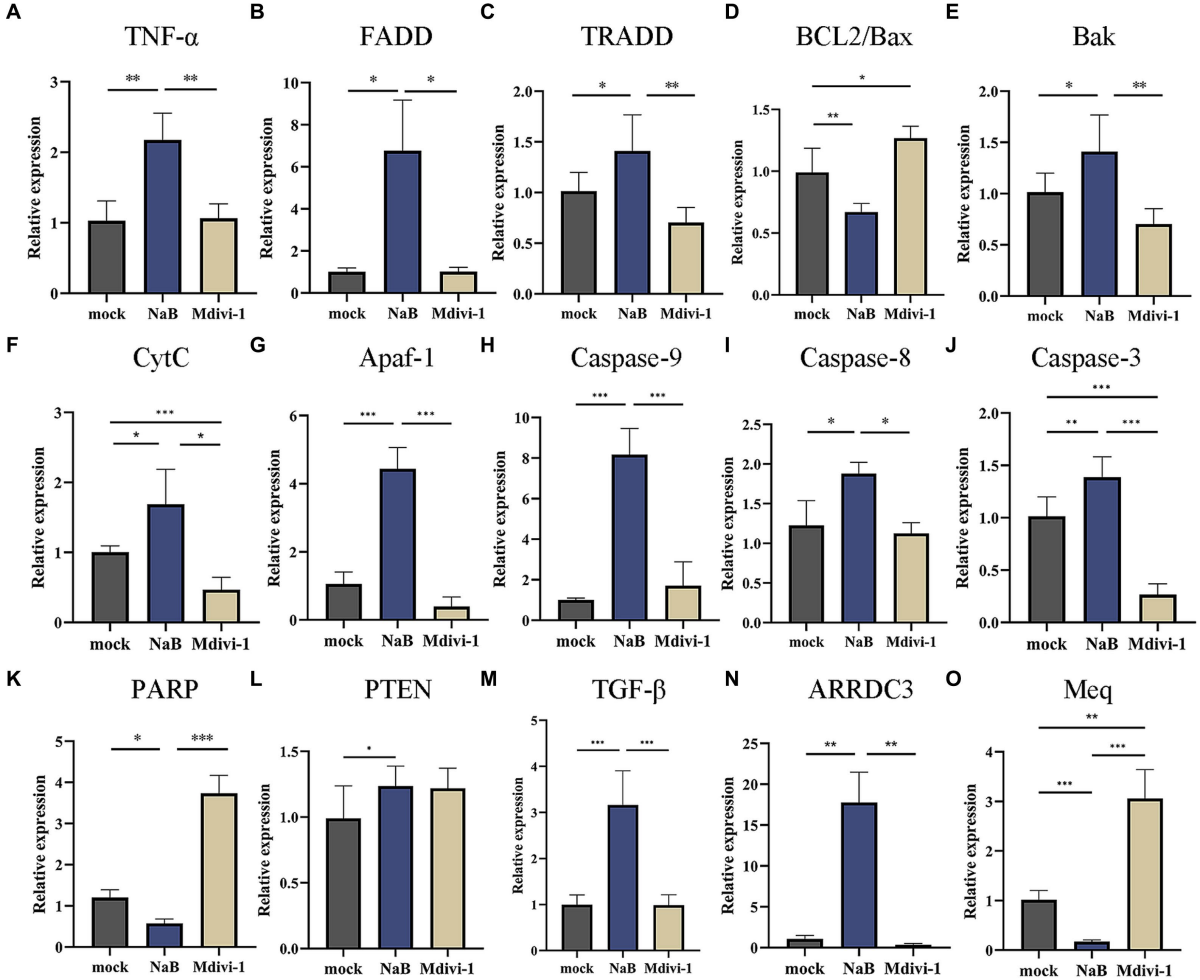
Expression of molecules in mitochondrial apoptosis signaling pathway and tumor related genes. **(A–K)** Relative expression of mitochondrial apoptosis signaling pathway related genes. **(L–N)** Relative expression of tumor suppressive genes. **(O)** Relative expression of oncogene *Meq*. *, * *, ***: statistically significant at *p* < 0.05, *p* < 0.01, *p* < 0.001 between groups, respectively.

To elucidate the molecular mechanism underlying the induction of apoptosis in MSB-1 cells by NaB, we assessed alterations in the transcriptional levels of cytokines associated with the mitochondrial apoptotic pathway. As a control, we incorporated Mdivi-1, a known inhibitor of mitochondrial outer membrane permeabilization that consequently retards apoptosis (45). The experimental framework comprised three distinct groups: NaB, mock, and Mdivi-1. The qPCR analysis revealed that NaB significantly enhanced the expression of *TNF-α*, *FADD*, and *TRADD* (Figures 10A–C). Additionally, we observed a decrease in the *BCL2*/*Bax* ratio in the NaB group, with the former being an anti-apoptotic protein and the latter a pro-apoptotic protein (Figure 10D). Furthermore, compared to the control group and Mdivi-1 group, the NaB group exhibited a significant increase in the activity of *Bak*, *CytC*, *Apaf-1*, *Caspase-9*, *Caspase-8*, Caspase*-3*, and decreased expression of *PARP* in MSB-1 cells (Figures 10E–K).

### 3.12 Effect of NaB on tumor suppressive genes in MSB-1

The expression of several tumor suppressive genes, such as *PTEN*, *ARRDC3*, and *TGF-β*, was investigated in MSB-1 cells after 24 h of NaB treatment. *PTEN*, an extensively studied tumor suppressor (46), plays a crucial role in tumor suppression. Down-regulation of *ARRDC3* promotes tumor cell proliferation and inhibits apoptosis (47). In the early stages of tumor progression, *TGF-β* acts as a tumor suppressor (48). Our experimental results demonstrated that NaB enhances the expression of these three tumor suppressive genes mentioned above. Therefore, it was postulated that NaB may hinder tumor progression by stimulating the expression of anti-tumor genes (Figures 10L,N). The oncogene *Meq*, encoded by MDV, plays a critical role in lymphocyte transformation (49). Notably, our experimental findings indicate that NaB substantially inhibits *Meq* expression (Figure 10O).

## 4 Discussion

In this study, utilizing MD5-infected chickens as our experimental model, we have shown that NaB possesses the ability to impede the replication of MDV via inhibiting the expression of immediate early (IE) genes *ICP4* and *ICP27*, which are crucial for lytic infection of MDV (50). Following an initial burst of lytic infection, T-lymphocytes become latently infected with MDV (51). Subsequently, chickens infected with virulent strain of MDV develop aggressive T-cell lymphomas (52). Notably, the majority of transcripts found in MDV lymphoblastoid cells are derived from IE genes (53), suggesting that these genes may play a significant role in the maintenance of latency (51). Our experimental findings provide compelling evidence that NaB shows a remarkable ability to reduce the morbidity of lymphomas in chickens following Md5 infection. Furthermore, NaB exhibits a significant capacity to suppress the oncogene *Meq* and upregulates the tumor suppressive genes such as *Meq* and *ARRDC3* in spleen, thereby positively influencing anti-tumor performance. The inhibitory effect of NaB on lymphomas may be attributed to its inhibition of IE genes, including *ICP4* and *ICP27*. Accumulated evidence to date suggests that *ICP4* may be associated with lytic infection and latency, with latently infected lymphocytes contributing to 75% of lymphoma (54). Latent infection of T-lymphocytes is a prerequisite to malignant transformation and neoplastic disease (55). However, the mechanism underlying MDV-induced latency and oncogenesis remains poorly understood.

**Figure 11:**
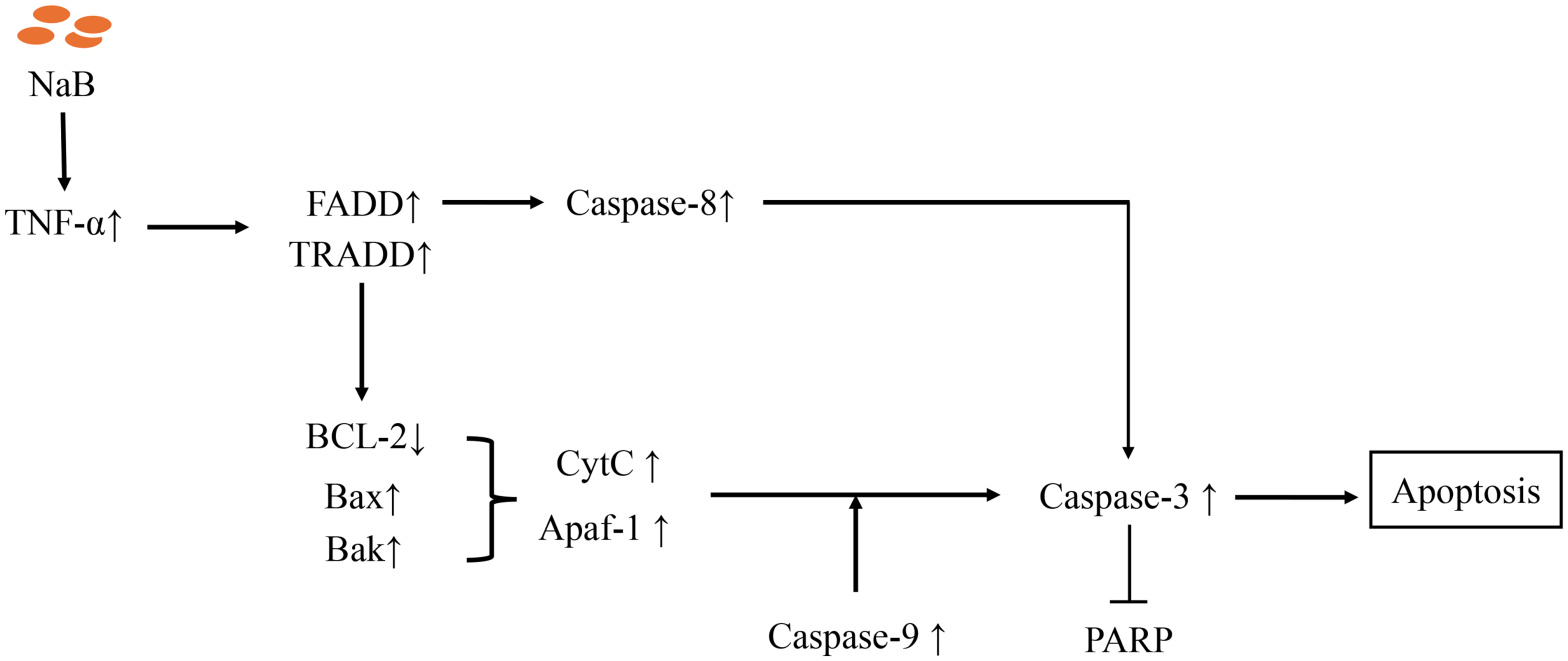
Molecular mechanisms of apoptosis mediated by Sodium Butyrate.

Apoptosis plays a pivotal role in the regulation of physiological growth and maintenance of tissue homeostasis. Our *in vivo* and *in vitro* experiments have shown that NaB can lead to apoptosis in MDV induced lymphomas cells. The apoptotic pathways encompass two major categories: endogenous and exogenous pathways (56). The endogenous pathway, known as the mitochondrial pathway, is a highly conserved mechanism of cell death that is indispensable for the development and sustenance of multicellular organisms (57). Our research further found that, in contrast to the mitochondrial apoptosis signaling pathway inhibitor Mdivi-1, NaB exhibits the ability to enhance the expression of the pivotal molecules in the mitochondrial signaling pathway, such as TNF*-α*, *FADD*, *TRADD*, *BCL2*, *Bax*, *CytC*, *Apaf-1*, *Caspase-8/9/3* etc. Intriguingly, our experimental findings are consistent with previous reports. Numerous investigations have unveiled the inhibitory effects of NaB on tumor cell proliferation and its ability to promote apoptosis both *in vitro* and *in vivo*. For instance, effectiveness has been demonstrated against prostate cancer cells DU145 and PC3 (58), lung cancer cells A549 (59), and gastric cancer cells GC (60). Alterations in mitochondrial permeability are central to the initiation of the mitochondrial apoptotic pathway, which involves the interconnected activation of Caspase molecules and pro-apoptotic members of the BCL family (61). Furthermore, the delicate equilibrium between pro- and anti-apoptotic BCL2 proteins determines the propensity of tumor cells to undergo apoptosis (62). Additionally, our experiments have elucidated that stimulation of NaB in MSB-1 cells can attenuate the *BCL2* to *BAX* ratio, consequently inducing apoptosis. These results suggest that NaB can achieve tumor cell apoptosis by regulating the mitochondrial apoptosis signaling pathway. The model of molecular mechanism is depicted in Figure 11, which expands the role of NaB in avian tumor.

Presently, vaccination serves as the primary approach for managing MD in chickens. However, despite the widespread utilization of the CVI988 vaccine in poultry flocks, MD cases continue to be reported in various regions in recent years (63, 64). Some investigations have raised concerns regarding the potential of the CVI988 vaccine to promote viral virulence (27) and recombination between vaccine strains and field strains (65). Given the absence of pharmacological treatments for avian MD tumors, exploring the potential of combining pharmacological interventions with vaccination could prove beneficial. Our data suggested the NaB is a potential prophylactic candidate which could be further investigated in the field of immunosuppressive neoplasia in chickens. Further investigation is warranted to delve into the implementation of NaB in the realm of practical production, with a specific emphasis on its convenience, cost-effectiveness, and efficacy in impeding the progression of MDV.

Consequently, our data showed that NaB has the potential to be incorporated into chicken lymphoma prevention and treatment regimens and contribute to the study of viral diseases in animals. We propose the consideration of incorporating an appropriate dosage of sodium butyrate into standard chicken diets as a potential approach to alleviate the clinical challenges attributed to MD.

## 5 Conclusion

In conclusion, our experiments establish that NaB exerts a potent inhibitory effect on lymphoma caused by MDV engaging the mitochondrial apoptotic pathway. Consequently, the utilization of NaB, a histone deacetylase inhibitor, warrants serious contemplation as a promising avenue for MDV prevention.

## Data availability statement

The raw data supporting the conclusions of this article will be made available by the authors, without undue reservation.

## Ethics statement

The animal study was approved by Ethics Committee for Animal Experiments of Foshan University. The study was conducted in accordance with the local legislation and institutional requirements.

## Author contributions

QL: Data curation, Methodology, Writing – original draft, Writing – review & editing, Formal Analysis, Software. JZ: Data curation, Methodology, Writing – review & editing, Validation. FY: Data curation, Methodology, Validation, Writing – review & editing, Investigation. CZ: Investigation, Writing – review & editing, Formal Analysis, Software. MC: Writing – review & editing, Methodology, Validation. CC: Validation, Writing – review & editing, Investigation. SC: Validation, Writing – review & editing. FW: Writing – review & editing. NW: Writing – review & editing. YC: Writing – review & editing. LQ: Writing – review & editing, Conceptualization, Funding acquisition, Supervision.
