# Supplementary material for: Sodium butyrate impedes the lymphoma caused by Marek’s disease virus via regulating the mitochondrial apoptosis pathway
Source: Front Vet Sci. 2024 Feb 28;11:1360878. doi: 10.3389/fvets.2024.1360878 (PMC10932986; doi:10.3389/fvets.2024.1360878)
Supplement: Supplementary file 1 [file Data_Sheet_1.docx]

**Biosecurity Measures**

The following are the biosecurity measures we took during the experiment:

Restricted Access: Limit access to areas where infected animals are housed to authorized personnel only. Visitors should be restricted, and proper biosecurity measures should be in place, including hand hygiene and wearing appropriate protective clothing.

Record Keeping: Maintaining detailed records of all activities related to virus handling, including sample collection, testing, and disposal.

Animal Isolation: Keep animals infected with non-human infectious viruses separate from healthy animals to prevent transmission. This can involve housing infected animals in dedicated isolation areas or separate facilities.

Sanitation and Disinfection: Regular cleaning and disinfection of animal housing, equipment, and surfaces are crucial to prevent the persistence and spread of the virus. Effective disinfectants should be used according to recommended protocols.

Sample collection: After completing the experiments, we package the animal carcasses in specialized plastic bags and store them in a freezer after disinfection (while also filling out and attaching animal carcass labels). These carcasses are regularly collected by institutions holding permits for the disposal of animal carcasses and waste, ensuring timely and proper disposal. We ensure the proper disposal of waste fluids, solid materials, animal carcasses, and specimens generated during the animal experimentation process.

Waste Management: Proper disposal of bedding, manure, and other waste materials from infected animals is essential. Animal bedding, feces, discarded feed, used syringes, cotton balls, gauze, and other waste materials are disposed of in yellow medical waste bins and treated with pressure steam sterilization. Various waste materials or fluids are discarded according to regulations within the experimental animal area.

According to the regulations established by the Ethics Committee for Animal Experiments of Foshan University, we adhere to a well-defined protocol for the proper disposal of experimental animals following euthanasia. Upon euthanasia, the animals are carefully placed in plastic bags within the laboratory premises, and responsibly assigned personnel then collect and sent to specialized third party institutions for harmless disposal.

Regular Audits and Inspections: Conducting regular audits and inspections of biosecurity protocols, equipment, and facilities helps ensure ongoing compliance and identify areas for improvement.
